# Status of dental caries and associated factors in Tibetan adults: findings from the fourth China National Oral Health Survey

**DOI:** 10.1186/s12903-020-01225-0

**Published:** 2020-09-07

**Authors:** Lingxia Guan, Jing Guo, Jinghao Ban, Gang Li, Juan Tong, Aiyun Chuan, Tian Tian, Bing Han, Kun Xuan, Shengchao Wang

**Affiliations:** 1 State Key Laboratory of Military Stomatology & National Clinical Research Center for Oral diseases & Shaanxi Key Laboratory of Stomatology, Department of Preventive Dentistry, School of Stomatology, The Fourth Military Medical University, 145 Changle West Road, Xi’an, Shaanxi 710032 China; 2 State Key Laboratory of Military Stomatology & National Clinical Research Center for Oral diseases & Shaanxi Key Laboratory of Stomatology, Department of Operative Dentistry and Endodontics, School of Stomatology, The Fourth Military Medical University, 145 Changle West Road, Xi’an, Shaanxi 710032 China

**Keywords:** Caries, Prevalence, Observational study, Adult

## Abstract

**Background:**

Tibet, a region where average elevation is above 3500 m and socio-economic development is relatively lower, was not included in National Oral Health Survey over decades. The cross-sectional study aimed to investigate the status of dental caries and associated factors in Tibetan adults.

**Methods:**

Participants aged 35–44, 55–64 and 65–74 years were selected. Decayed, missing, and filled tooth (DMFT), decayed and filled root (DF-Root) and root canal index (RCI) were used to evaluate dental caries. Questionnaire survey on demographic information, socioeconomic status, dietary habits, and oral health knowledge and behavior was conducted. Mann-Whitney U test, logistic regression were used for the statistical analyses.

**Results:**

A total of 446 participants were enrolled in the survey. Of these: 222 (49.8%) were females, 224 (50.2%) were males; 149 (33.4%), 151 (33.9%), 146 (32.7%) were aged 35–44, 55–64 and 65–74 years respectively. The mean DMFT (SD) was 7.62 (4.84), 12.46 (8.16), and 21.38 (8.93). The filling rate was very low in all age groups (1.77%, 0.98%, 0.45%). The mean DF-Root (SD) was 0.50 (1.04), 1.04 (2.02), 1.32 (2.14), respectively. Root caries index was 42.27, 44.78 and 57.60%. Older age (65–74 age group) was positively associated with crown caries (odds ratio = 31.20, 95% confidence interval: 10.70–90.96). College degree and above and brushing teeth at least once a day were negatively associated with crown caries (odds ratio = 0.28, 95% confidence interval: 0.09–0.89; odds ratio = 0.39, 95% confidence interval: 0.21–0.72, respectively). Rural area, high income level and brushing teeth at least once a day were negatively and tooth with attachment loss was positively associated with root caries.

**Conclusions:**

The status of dental caries in the adults in Tibet is severe and the treatment rate is very low. The study suggests a correlation between crown caries and the variables age, level of education and frequency of tooth brushing; correlation between root caries and residence, income level, frequency of tooth brushing and exposed root surfaces. These findings could be as reference to develop community based interventions to reduce the prevalence of caries in Tibet.

## Background

Dental caries is the localized destruction of susceptible dental hard tissues by acidic by-products from bacterial fermentation of dietary carbohydrates [1], which ranked among the ten most prevalent chronic conditions [2]. Despite the widespread decline in caries prevalence and severity in permanent teeth in high-income countries over the past few decades [3, 4], the prevalence and burdens of caries are heavy over the world [5, 6]. In the United States (US), the 2013–2014 National Health Nutrition and Examination Survey showed that 33.4% of adults aged 21–64 years old had untreated coronal dental caries [7]. A study on the prevalence of root caries in a sample of Japanese elderly people showed that 39% of participants had one or more decayed roots and 53% had at least one decayed or filled lesion [8]. In Guangdong province of China, an epidemiological investigation conducted in 2015–2016 showed that the prevalence of caries on crown among age groups of 34–44, 55–64 and 65–74 years was 71.18, 77.08 and 81.2%, respectively, and the prevalence of root caries was 28.47, 59.38 and 63.19% [9, 10]. Facing with the high prevalence and burdens worldwide, caries is the primary cause of oral pain and tooth loss [11], and thus significantly affect the general health and quality of life of people globally [2, 12].

Although globally recognized success has been achieved, China still confronts numerous problems, such as inequalities in healthcare resources across regions and inefficiency of the medical service delivery in certain areas [13, 14]. Conducting regular oral health surveys has become increasingly important as a public health surveillance measure. National and regional data from regular oral health surveys are useful to assess oral health and needs, explore disparities between regions, and plan intervention programs and policies at national and local levels [15, 16]. In the National Oral Health Survey conducted between 2015 and 2016, Tibet was included for the first time.

Therefore, our primary objectives were to 1) determine the status of dental caries of adults in Tibet and 2) identify the associated factors in the Tibetan adult population.

## Methods

### Study design and sample selection

According to the recommendation by the World Health Organization (WHO), age groups of 35–44 and 65–74 years are representative adult age groups to check oral health in a large population [17]. In order to observe the developmental trends of oral disease, the present survey included additional 55–64 age group. Therefore, the current study evaluated the oral health status among three age groups of 35–44, 55–64, and 65–74 years.

The analysis included data from participants aged 35–44, 55–64 and 65–74 years old in Tibet. With a population of 3.08 million in 2012, Tibet is located in southwest of China and covers an area of 1,202,230 km^2^, accounting for one eighth of Chinese total land mass, and ranking second in China. Tibet suffers from lower level of economic development and rare distribution of health care resources.

**Fig. 1:**
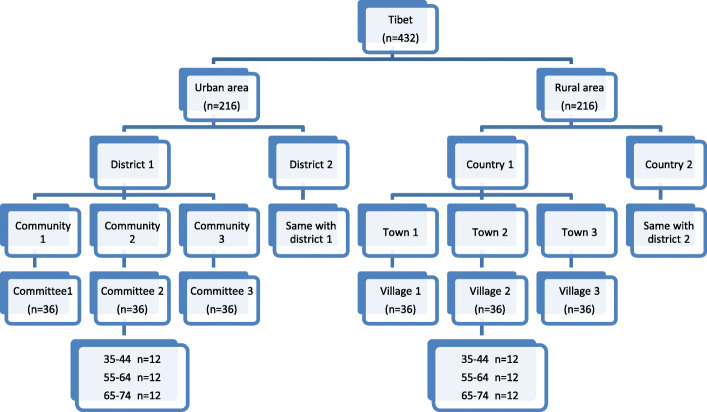
A multistage stratified random sampling technique of the present study used per the overall design of the fourth China National Oral Health Survey

The cross-sectional study was conducted in 2015–2016 by the Fourth Military Medical University, China. A multistage stratified random sampling technique was employed (Fig. 1). In the first stage, two districts including Lhasa and Xigaze and two countries including Nyingchi and Nagqu were chosen randomly using the Probability Proportional to size (PPS) method. In the second stage, three neighborhood committees in each district and three village committees in each country were selected randomly. In the third stage, participants were recruited by quota sampling [18]. Our study was aim to determine the DMFT values in different age groups (35–44, 55–64 and 65–74) and areas (Urban and Rural) in the cross-sectional survey. The NCSS PASS 11 was use to calculated the sample size. A sample size of 64 in each group should produce a two-sided 95% confidence interval with the precision that is equal to 2.5 when the estimated standard deviation is 10.0 (from the third National Epidemiological Survey of Oral Health) [19]. The minimum sample size was 64 for each age group in urban or rural areas. The study protocol was approved by the Stomatology Ethics Committee of Chinese Stomatological Association (Approval no. 2014–003). Informed consent was obtained from each study participant before enrollment.

### Quality control

Before the survey, four examiners and two investigators (all registered dentists) were trained by the fourth China National Oral Health Survey committee to ensure the validity and reliability of the results [18]. All examiners and investigators were requested to pass the standard test before they were certified for the survey (kappa≥0.80). During the survey, 5% of the participants were randomly selected for a second examination every day to test the inter-rater reproducibility.

### Clinical examination

According to criteria recommended by the WHO and the fourth National Oral Health Survey, uniform devices and equipment including plane mouth mirrors, tweezers, and Community Periodontal Index (CPI) periodontal probes were used to evaluate the caries status of both crown and root of 32 teeth [16, 17]. Portable dental chairs equipped with artificial lights were used in our survey.

Decayed, missing, and filled tooth (DMFT), comprised of DT, MT and FT, were indices to evaluate the severity of crown caries. Decayed and filled root (DF-Root), comprised of D-Root and F-Root, was used to assess the severity of root caries. Root caries index (RCI) (number of teeth with root caries lesions/ number of teeth with attachment loss) was used to describe the risk of root caries [20]. Caries prevalence (percent of participants whose DMFT > = 1 or DF-Root > = 1 to the total number of participants) [20]. Crown caries was recorded when obvious cavity or undermined enamel cavity was observed by visual inspection or detectably softened floor was confirmed by the CPI probe. Root caries was measured when soft leather-like lesion was found on exposed root using the CPI probe. Crown and root caries were recorded separately. If a lesion affected both crown and root, crown and root caries were both recorded [20]. And clinical attachment loss (CAL) which may be associated with root caries was also detected using CPI probe.

### Questionnaire survey

During the face-to-face interviews, a structured questionnaire (Supplementary file 1 and 2) was used by trained interviewers to collected information on demographic factors (age, gender [male or female], residence [urban or rural]), socio-economic factors (education levels [illiteracy and primary school, middle school, college degree and above] [21], annual household income [≤20,000 RMB, 20,000- < 40,000 RMB, ≥40,000 RMB, 20000 RMB is approximately US$2800] [22], occupation), dietary habits (frequency of dessert [scarce, <once/day, ≥once/day], frequency of sugary beverage [scarce, <once/day, ≥once/day]) [23], as well as the knowledge, attitude and practice of oral health (frequency of tooth brushing [<once/day, ≥once/day], dental visit experience [yes, no], and knowledge and attitude of oral health [positive (<median score), negative (≥median score)] [24]). In the questionnaire of our study, 12 questions regarding attitude and knowledge of oral heath were involved. These questions included attitude toward importance of oral health, regular oral examination, relationship of oral disease and self protection, importance of oral disease prevention and knowledge about gingival bleeding, caries, fluoride, pit and fissure sealants and so on. Positive attitude and knowledge means answer these questions correctly > = 6, and negative attitude and knowledge means answer these questions correctly < 6.

### Data analysis

Descriptive analysis was used to present the mean values of DMFT, DF-Root, caries prevalence and RCI. Due to the skewed distribution of continuous variables, non-parametric test (Mann-Whitney U-test) was used to compare the mean DMFT and DF-Root between two gender groups or two resident groups. Crown caries was categorized as binary variables (severe vs. moderate) using75^th^ percentile as cut-off (DMFT: <vs. ≥ 21) [25]. Root caries was dichotomized using DF-root: <vs. ≥1. The univariate and multiple logistic regression were applied for the associated factor analysis for the high DMFT and DF-root value with the Survey logistic procedure. All statistical analyses were done using SAS9.4 (SAS Institute, Cary, NC, USA).

## Results

A total of 446 were enrolled in the survey and response rate was 100%. Of these, 149, 151 and 146 were aged 35–44, 55–64 and 65–74 years respectively. The majority were Tibetan (433 [97.1%]), while the rest were Han ethnicity (10 [2.2%]) and other ethnic minorities (3[0.7%]).

### Crown caries of Tibetan in three age groups

DMFT index and its components, Prevalence of caries, and filling rate are presented in Table 1. The prevalence of caries in three age groups was 98, 98 and 100%, respectively, and no difference was found between males and females and also between urban and rural. The mean DMFT (SD) index of three age groups was 7.62 (4.84), 12.46 (8.16), 21.38 (8.93), and it was higher in females than in males in the age group of 35–44 years (*P* < 0.05), and higher in rural area than urban area in the age group of 65-75 years (*P* < 0.05). The filling rates were 1.77%, 0.98 and 0.45%, respectively.

**Table 1:** Crown caries status of three age groups in Tibet, 2015–2016

Age group	Variables	*n*	%	DMFT		D			M			F			Filling rate (%)
				Mean	SD	Mean	SD	Rate (%)	Mean	SD	Rate (%)	Mean	SD	Rate (%)	
35–44	Male	71	97.2	6.65*	4.43	3.37*	2.93	50.64	3.17	2.48	47.67	0.11	0.36	1.69	3.24
	Female	78	98.7	8.50	5.05	5.47	3.98	64.40	2.97	2.43	34.99	0.05	0.27	0.60	0.92
	Urban	78	97.1	7.72	4.59	4.45	3.23	57.64	3.12	2.61	40.37	0.15*	0.43	1.99	3.34
	Rural	71	98.8	7.51	5.12	4.49	4.11	59.85	3.01	2.27	40.15	0.00	0.00	0.00	0
	Total	149	98.0	7.62	4.84	4.47	3.66	58.68	3.07	2.45	40.26	0.08	0.32	1.06	1.77
55–64	Male	73	97.3	11.19	8.59	4.77	5.48	42.59	6.63	6.81	57.04	0.04	0.20	0.37	0.85
	Female	78	98.7	13.65	7.60	5.59	4.71	43.29	7.68	5.96	56.24	0.06	0.30	0.47	1.07
	Urban	73	98.6	12.82	8.18	5.73	5.15	44.66	7.01	6.16	54.70	0.08	0.32	0.64	1.42
	Rural	78	97.4	12.13	8.18	5.01	5.08	41.33	7.09	6.65	58.46	0.03	0.16	0.21	0.51
	Total	151	98.0	12.46	8.16	5.36	5.11	42.99	7.05	6.40	56.59	0.05	0.25	0.43	0.98
65–74	Male	80	100	21.05	8.71	3.37*	3.78	16.03	17.65	9.31	83.85	0.03	0.22	0.12	0.74
	Female	66	100	21.79	9.24	5.86	5.46	26.91	15.91	9.37	70.02	0.02	0.12	0.07	0.26
	Urban	71	100	19.46*	9.11	4.97	4.40	25.54	14.48*	8.91	74.38	0.01	0.12	0.07	0.28
	Rural	75	100	23.20	8.42	4.05	5.07	17.47	19.12	9.23	82.41	0.03	0.23	0.11	0.65
	Total	146	100	21.38	8.93	4.50	4.76	21.04	16.86	9.34	78.86	0.02	0.19	0.10	0.45
Total		446	98.7	13.76	9.41	4.78	4.56	34.73	8.93	8.81	64.90	0.05	2.56	0.37	1.07

*P < 0.05when comparing means between male and female, or urban and rural

Abbreviations**:** *D* decayed tooth, *M* missing tooth, *F* filled tooth, *DMFT* decayed, missing, and filled tooth, *S.D.* standard deviation

**Fig. 2:**
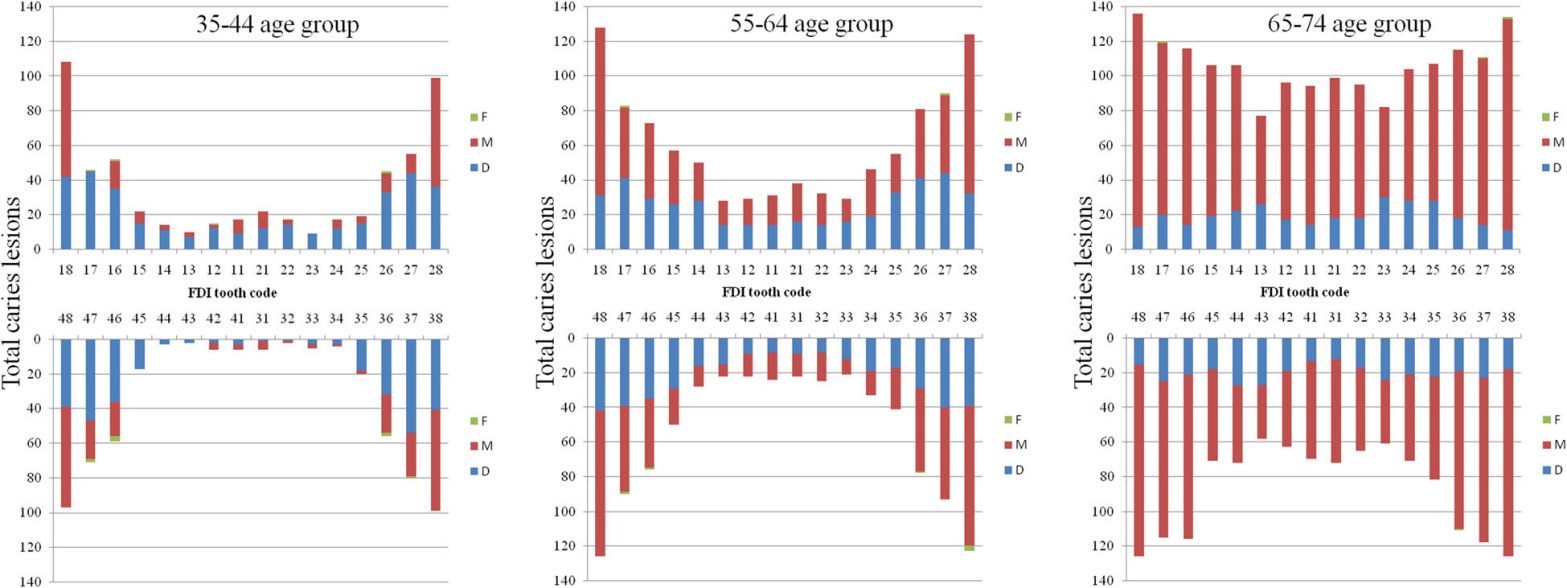
Distribution of DMFT on all 32 teeth

Figure 2 shows the distribution of DMFT and three components (D, M and F) and on all 32 teeth in mandibular and maxillary aches. In the age groups of 35–44 and 55–64 years, DMFT, D and M were mostly concentrated in molars. Anterior teeth, especially canine, experienced the fewest numbers of DMFT, D and M. Number of DMFT in maxillary was higher than in mandibular. In the age group of 65–74 years, no obvious difference of DMFT number between posterior teeth and anterior teeth was found. An increasing tread between age and numbers of DMFT was observed.

### Root caries of Tibetan in three age groups

The prevalence of root caries, DF-Root index and components, and RCI of different genders and residents in three age groups are shown in Table 2. The respective prevalence of root caries was 27.5,39.7, and 49.3%, and no difference was found between males and females and between urban and rural area. The DF-Root (SD) index of three age groups was 0.50 (1.04), 1.04 (2.02), and 1.32 (2.14). Among all participants, only one participant filled the root caries. RCI of three age groups was 42.27%, 43.94, and 57.60%, respectively, which was higher in the age group of 65–74 years than other two groups.

**Table 2:** Root caries status of three age groups in Tibet, 2015–2016

Age group	Variables	*n*	%	DF-Root		D-Root		Rate (%)	F-Root		Rate(%)	RCI (%)
				Mean	SD	Mean	SD		Mean	SD		
35–44	Male	71	28.2	0.52	1.15	0.52	1.15	100	0.00	0.00	0	38.46
	Female	78	26.8	0.49	0.94	0.49	0.94	100	0.00	0.00	0	46.67
	Urban	78	28.2	0.54	1.07	0.54	1.07	100	0.00	0.00	0	43.13
	Rural	71	26.8	0.46	1.01	0.46	1.01	100	0.00	0.00	0	41.30
	Total	149	27.5	0.50	1.04	0.50	1.04	100	0.00	0.00	0	42.27
55–64	Male	73	41.1	1.11	2.33	1.11	2.33	100	0.00	0.00	0	44.12
	Female	78	38.5	0.97	1.69	0.97	1.69	100	0.00	0.00	0	46.88
	Urban	73	42.5	1.03	1.70	1.03	1.70	100	0.00	0.00	0	46.97
	Rural	78	37.2	1.05	2.29	1.05	2.29	100	0.00	0.00	0	43.94
	Total	151	39.7	1.04	2.02	1.04	2.02	100	0.00	0.00	0	44.78
65–74	Male	80	48.8	1.03	1.44	1.01	1.44	98.78	0.01	0.11	1.22	60.00
	Female	66	50.0	1.67	2.73	1.67	2.73	100	0.00	0.00	0	55.00
	Urban	71	53.5	1.37	1.94	1.37	1.94	100	0.00	0.00	0	61.29
	Rural	75	45.3	1.27	2.32	1.25	2.32	98.95	0.01	0.12	1.05	53.97
	Total	146	49.3	1.32	2.14	1.31	2.14	99.48	0.01	0.08	0.52	57.60
Total		446	38.8	0.95	1.83	0.95	1.83	99.76	0.00	0.05	0.24	48.87

*P < 0.05when comparing means between male and female, or urban and rural

Abbreviations**:** *D-Root* decayed root, *F-Root* filled root, *DF-Root* decayed and filled root, *S.D.* standard deviation, *RCI* root caries index

### Factors associated with dental caries severity

In the univariate analysis (Tables 3 and 4), older age (65–74 age group) (OR = 51.96, 95% CI: 18.242–147.98), brushing teeth at least once a day (OR = 0.24, 95% CI: 0.15–0.37), positive knowledge and attitude of oral health (OR = 1.75, 95% CI: 1.14–2.68) had significant associations with crown caries. Older age (65–74 age group) (OR = 2.56, 95% CI: 1.58–4.16), high level of education (OR = 0.35, 95% CI: 0.17–0.73), high level of income (OR = 0.43, 95% CI: 0.27–0.68), brushing teeth at least once a day (OR = 0.51, 95% CI: 0.34–0.76), positive knowledge and attitude of oral health (OR = 1.61, 95% CI: 1.10–2.36), tooth with attachment loss (OR = 4.54, 95% CI: 2.48–8.33) had significant associations with root caries.

**Table 3:** Univariate and multiple logistic regression analysis for the high DMFT value of the crown

Factors	Categories	DMFT value		Univariate logistic regression		Multiple logistic regression	
		Cases (DMFT≥21), n	Control (DMFT< 21), n	Unadjusted OR (95%CI)	*P*	Adjusted OR ^a^ (95%CI)	*P*
Gender	Male	58	166	1	0.79	1	0.42
	Female	60	162	1.06 (0.70–1.62)		1.26 (0.72–2.20)	
Residence	Urban	51	171	1	0.1	1	0.78
	Rural	67	157	0.70 (0.46–1.07)		1.11 (0.54–2.27)	
Age	35–44	4	145	1	–	1	–
	55–64	28	123	8.25 (2.82–24.17)	0.69	5.11 (1.67–15.64)	0.81
	65–74	86	60	51.96 (18.242–147.98)	< 0.0001	31.20 (10.70–90.96)	< 0.0001
Level of education	Low (Illiteracy and primary school)	116	245	1	–	1	–
	Medium (Middle school)	2	33	0.13 (0.03–0.54)	0.96	0.47 (0.06–3.43)	0.31
	High (College degree and above)	0	50	< 0.001 (< 0.001- > 999.99)	0.95	0.28 (0.09–0.89)	< 0.0001
Level of income	Low (≤ 20,000 RMB)	81	175	1	–	1	–
	Medium (20,000- < 40,000 RMB)	14	47	0.64 (0.34–1.24)	0.85	1.27 (0.56–2.89)	0.66
	High (≥ 40,000 RMB)	23	106	0.47 (0.28–0.79)	0.06	0.71 (0.36–1.41)	0.33
Frequency of tooth brushing	Low (< once/day)	70	84	1	< 0.0001	1	0.006
	High (≥ once/day)	48	244	0.24 (0.15–0.37)		0.39 (0.21–0.72)	
Dental visit experience	Yes	57	160	1	0.93	1	0.51
	No	61	168	1.02 (0.67–1.55)		0.78 (0.42–1.47)	
Knowledge and attitude of oral health	Positive	47	176	1	0.01	1	0.21
	Negative	71	152	1.75 (1.14–2.68)		0.72 (0.39–1.33)	
Frequency of dessert	Scare	78	180	1	–	1	–
	< once/day	29	118	0.57 (0.35–0.92)	0.08	0.64 (0.32–1.30)	0.45
	≥ once/day	11	30	0.85 (0.40–1.77)	0.76	2.75 (0.87–8.68)	0.28
Frequency of drink with sugar	Scare	99	216	1	–	1	–
	< once/day	15	93	0.35(0.19–0.64)	0.10	0.56 (0.24–1.29)	0.80
	≥ once/day	4	19	0.46(0.15–1.39)	0.65	0.40 (0.10–1.58)	0.39

^a^Adjusted for other factors

**Table 4:** Univariate and multiple logistic regression analysis for the high DF-root value

Factors	Categories	DF-root value		Univariate logistic regression		Multiple logistic regression	
		Cases (≥1), n	Control (=0), n	Unadjusted OR (95%CI)	*P*	Adjusted OR ^a^ (95%CI)	*P*
Gender	Male	89	135	1	0.68	1	0.71
	Female	84	138	0.92 (0.63–1.35)		0.92 (0.60–1.41)	
Residence	Urban	91	131	1	0.34	1	0.04
	Rural	82	142	0.83 (0.57–1.22)		0.59 (0.35–0.98)	
Age	35–44	41	108	1	–	1	–
	55–64	60	91	1.74 (1.07–2.82)	0.69	1.26 (0.74–2.15)	0.92
	65–74	72	74	2.56 (1.58–4.16)	0.001	1.52 (0.84–2.78)	0.23
Level of education	Low (Illiteracy and primary school)	150	211	1	–	1	–
	Medium (Middle school)	13	22	0.83 (0.41–1.70)	0.39	1.23 (0.56–2.71)	0.36
	High (College degree and above)	10	40	0.35 (0.17–0.73)	0.02	0.73 (0.30–1.78)	0.34
Level of income	Low (≤ 20,000 RMB)	114	142	1	–	1	–
	Medium (20,000- < 40,000 RMB)	26	35	0.93 (0.53–1.63)	0.22	1.08 (0.58–2.01)	0.20
	High (≥ 40,000 RMB)	33	96	0.43 (0.27–0.68)	0.001	0.52(0.31–0.88)	0.01
Frequency of tooth brushing	Low (< once/day)	76	78	1	0.001	1	0.009
	High (≥ once/day)	97	195	0.51 (0.34–0.76)		0.51 (0.31–0.84)	
Dental visit experience	Yes	93	124	1	0.09	1	0.11
	No	80	149	0.72 (0.49–1.05)		0.69(0.44–1.09)	
Knowledge and attitude of oral health	Positive	74	149	1	0.02	1	0.12
	Negative	99	124	1.61 (1.10–2.36)		1.46 (0.91–2.36)	
Frequency of dessert	Scare	101	157	1	–	1	–
	< once/day	59	88	1.04 (0.69–1.58)	0.40	1.12 (0.67–1.86)	0.78
	≥ once/day	13	28	0.72 (0.36–1.46)	0.32	1.06 (0.48–2.35)	0.99
Frequency of drink with sugar	Scare	126	189	1	–	1	–
	< once/day	40	68	0.88 (0.56–1.39)	0.78	0.82(0.48–.42)	1.00
	≥ once/day	7	16	0.66 (0.26–1.64)	0.44	0.68 (0.250–1.84)	0.56
Loss of attachment	No	14	78	1 4.54 (2.48–8.33)	< 0.001	1	< 0.001
	Yes	159	195			3.94 (2.10–7.39)	

^a^Adjusted for other factors

In the multivariate logistic regression analysis (Tables 3 and 4), older age (65–74 age group) (OR = 31.20, 95% CI: 10.70–90.96) was positively associated with crown caries while high level of education (OR = 0.28, 95% CI: 0.09–0.89) and brushing teeth at least once a day (OR = 0.39, 95% CI: 0.21–0.72) were negatively associated with crown caries. Rural area (OR = 0.59, 95% CI: 0.35–0.98), high level of income (OR = 0.52, 95% CI: 0.31–0.88), brushing teeth at least once a day (OR = 0.51, 95% CI: 0.31–0.84) were negatively associated with root caries while teeth with attachment loss (OR = 3.94, 95% CI: 2.10–7.39) was positively with root caries.

## Discussion

To the best of our knowledge, the current study investigated the caries status and associated factors among three adult age groups (35–44, 55–64 and 65–74) in Tibet for the first time. We found higher DMFT values in Tibet than those in all China. However, the rate of filling cases was very low in all age groups. Root caries prevalence was not as high as estimated. Older age and frequency of brushing teeth were common significant associated factor both in crown and root caries.

The DMFT index of three age groups was much higher than that observed in the fourth National Oral Health Survey (4.54, 8.69 and 13.33) [24] and a separate study conducted in Sichuan province (DMFT index of 65–74 age group was5.13) [26], although it was lower than other developing countries, such as Chile (DMFT index of 35–44 age group was 15.06) [27]. The D + F index observed in Tibet was also higher than that reported in Liaoning province during 2015–2016 (3.37, 3.40 and 4.09) [28]. This indicated that dental caries is a common and highly-severe disease in Tibet. As the second largest province in China, however, Tibet has relatively backward industries, agriculture and farming. Thus Tibet is considered underserved with a much lower rate of disease prevention and treatment than those in other areas [29]. In addition, high altitude and lack of oxygen in Tibet may have led to the reduction of secretary immunoglobulin A (SIgA) [30], a widely existing nonspecific immune protein in the tissue fluids and secretions that have shown to play an important role in the caries immune response [31–33] and have a negative correlation with caries disease [34–36]. Prior studies have found that people living in area with altitude > 3700 m have lower SIgA secretion than people from lower altitude [34–36]. Future studies are warranted to investigate this possible pathway and develop effective pharmacy or intervention measures to increase the SIgA levels.

The filling rate revealed that only 1.07% of teeth got treated, including many bad fillings. Most adults did not seek treatment for decayed teeth and only had some medicine to relieve pain. A prior study found that Tibet has the fewest number of active beds in China, with less than 3.3 beds per 1000 persons [37]. Given the lack of local medical resource, limited oral medical services were available to the patients.

DMFT index, especially D index, of females was higher than that of males in three age groups. Our observation was consistent with results of other studies in Malawi and northeastern China [38, 39], and suggests that females may have encountered severer problems in active caries than males. The observed gender difference may due to earlier eruption of teeth in females, hence longer exposure to the cariogenic oral environment and easier access to food supplies by women and frequent snacking during food preparation. And in many regions of Tibet, females suffered discrimination in access to medical resource. Other reasons for example lower flow rate of saliva in females than males also could resulted severe caries [40]. Another reason maybe that female are more likely to be affected by autoimmune diseases such as xerostomia and then cause dental caries [41].

In addition, the present survey showed that DMFT index in 65–74 age group of Tibet was higher in rural areas than that in the urban areas (*P* < 0.05). MT index in this age group accounted for 78.86% of DMFT, and was the main component caused difference of DMFT between rural and urban areas. Study has showed that rural residence is negatively associated with the frequency of dental care visits [42]. Most of the elderly individuals in rural areas prefer to remove a seriously decayed teeth rather than restoring it, leading to more MT. This situation may result from low income, limited access to dental care services, and limited healthcare knowledge.

The prevalence of root caries and DF-Root index among age group of 35–44 years in Tibet were similar to those reported in other parts of China. However, the two indices were much lower among age group of 65–74 years in Tibet (38.8% vs 61.9%, 1.32 vs 2.64) compared to other parts of China [20], and the difference could be the high rate of missing teeth in Tibet. In support of this statement, the M in the current study among age group of 65–74 years was much higher than that observed in other parts in China (16.86 vs. 9.50). Like the low filling rate of crown caries, the filling rate of root caries was also very low. Only one root caries was filled in the present study, which was much lower than the filing rate in other country [43]. This finding further illustrates that the oral health awareness and more medical resources needs to be strengthened in Tibet.

Older age and brushing teeth at least once a day were the common significant associated factors of crown and root caries. The association between age and crown caries was consistent with the result of first Uruguayan National Oral Health Survey [44]. According to the results in Table 1, missing teeth (component M) accounted majority of the difference among three age groups. Older adults probably experienced more dental caries over the course of life and extracted teeth according to their past needs and conditions. The high percent of tooth loss reflected the fact that the treatment has largely been extraction of the affected tooth instead of removing the least possible amount of decayed tooth in Tibet. After adjusted by other factors, significant difference in root caries caused by older age disappeared. According to Table 2, component D-Root accounted for majority of DF-Root. This outcome indicated that effect of older age on root caries appear to be mediated largely by other factors such as frequency of tooth brushing. Brushing teeth at least twice a day is a recommended oral health method, but in the present study only 61 adults answered brushing teeth twice a day or above in the questionnaire. So once a day was used as criteria in the study, and the same classification also was used in other previous research [24, 45]. Increasing the frequency of teeth brushing could be helpful to decrease the crown caries. Since brushing teeth is a simple and effective method to clean the plaque and reduce the risk of caries, it could be included in the intervention programs to prevent the dental caries.

Rural residence became a protector factor after adjusted by other factors in root caries. Table 1 has shown that the number of tooth loss in rural population was significantly higher than that in urban population, especially in the elderly group. That is to say, the number of residual teeth in rural population was less than that in urban population. So the root caries were correspondingly less in rural area than in urban areas.

The knowledge and skills attained through education may affect a person’s cognitive functioning, make them more receptive to health education messages, or better enable them to learn better oral health habit [46]. Meanwhile high level of education always was the indicator of high income. So the negatively effect of high level of education on crown caries may be covered by income level and frequency of brushing teeth.

In the unadjusted logistic regression analysis, the higher the household income, the lower was the prevalence of caries both in crown and root. Generally, higher household income allowing access to services, which may improve health directly (such as proper oral health preventive services and other treatment measures) or indirectly (such as behaviors) [46]. After adjusting for age group, educational level, frequency of tooth brushing, knowledge and attitude of oral health, the effects of income disappeared in crown. This result shows that the effects of income inequality on crown caries experience appear to be mediated largely by oral health-related behaviors.

Knowledge and attitude of oral health was important predictor for dental caries. People with positive knowledge and attitude of oral health usually had good habit about oral health. And this protective effect was mediated largely by other oral health-related behaviors.

Tooth with attachment loss was a meaningful predictor for root caries. The result was similar to Hayes’s study [45]. Exposed roots with poor plaque control created a favorable environment for root caries. But for attachment loss, a systematic review of 19 studies had not reached a consistent result [47], and further studies are warranted to validate our finding.

### Strengths and limitations

The current study investigated the prevalence of caries as well as the associated factors in Tibet for the first time. The study used credible method (PPS) for sampling and presented the caries data comprehensively by different age group, gender and residence area. However, we considered that reporting bias was existed in questionnaires of the present study. The majority of detected people were Tibetan and different comprehension of participants caused by language was inevitable. Additionally, in order to investigate the relationship between these discrete responses and a set of explanatory variables, multiple logistic regression was used to determine the impact that different variables have on caries, which may have limitation on interpreting the inter-relationship between the variables.

## Conclusion

In conclusion, the caries status of Tibetan people is severer compared to other parts of China; and decayed and missing teeth account for majority of DMFT values. At the same time, the filing rate was much lower in Tibet with only about 1–2% people had filled teeth. Therefore, it is urgent to increase the accessibility of medical resources in Tibet and strengthen the educations of oral health education in the region. Education such as increasing the frequency of tooth brushing, correct usage of dental floss and encouraging regular visits to dentists could be considered.

## Supplementary information

**Additional file 1: Supplementary file 1.** Questionnaire in Chinese.

**Additional file 2: Supplementary file 2.** Questionnaire translated into English.

## Data Availability

The datasets used and analyzed during the current study are available from the corresponding author on reasonable request.

## Abbreviations

D: Decayed tooth
M: Missing tooth
F: Filled tooth
DMFT: Decayed, missing, and filled tooth
D-Root: Decayed root
F-Root: Filled root
DF-Root: Decayed and filled root
S.D.: Standard deviation
RCI: Root caries index
PPS: Probability Proportional to size
CPI: Community Periodontal Index
