# Supplementary material for: Status of dental caries and associated factors in Tibetan adults: findings from the fourth China National Oral Health Survey
Source: BMC Oral Health. 2020 Sep 7;20:248. doi: 10.1186/s12903-020-01225-0 (PMC7487806; doi:10.1186/s12903-020-01225-0)
Supplement: Supplementary file 1 — Additional file 1: Supplementary file 1. Questionnaire in Chinese. [file 12903_2020_1225_MOESM1_ESM.pdf]

第四次中国口腔健康调查问卷（成人）（In Chinese）

被调查者 ID 号：□□□□□□□□□□□□□　　被调查者姓名：__________
调查日期：201□年□□月□□日　　　　调查员编号:□
要求：请在选择题相应选项前面的“□”内划“√”。

1. 您的最高学历是?（**只选一个答案**）
   1) □没有上过学　2) □小学　3) □初中　4) □高中
   5) □中专　6) □大专　7) □本科　8) □硕士及以上

2. 您平时进食以下食品或饮料的频率如何? **(每小题选一个答案)**

	6<br>每天<br>≥2 次	5<br>每天<br>1 次	4<br>每周<br>2-6 次	3<br>每周<br>1 次	2<br>每月<br>1-3 次	1<br>很少/<br>从不
1）甜点心（饼干、蛋糕、面包）及糖果（巧克力、含糖口香糖）	□	□	□	□	□	□
2）甜饮料（糖水、可乐等碳酸饮料，橙汁、苹果汁等果汁、柠檬水等非鲜榨果汁）	□	□	□	□	□	□
3）加糖的牛奶、酸奶、奶粉、茶、豆浆、咖啡	□	□	□	□	□	□

3. 您吸烟吗？（**只选一个答案**）
   1) □吸烟　2) □从不吸　3) □已戒烟（**选 2 或 3 项者不回答第 4、5 题**）

4. 您吸烟多少年了？ ____________年。（**请填一个整数，不知道或拒绝回答的填写“N”**）

5. 最近一个月内，您平均每天吸多少支烟？（**只选一个答案**）
   1) □　≤1 支/天　2) □　1-5 支/天　3) □　6-10 支/天
   4) □　11-20 支/天　5) □　21-40 支/天　6) □　≥41 支/天

6. 您喝白酒吗？（**只选一个答案**）
   1) □每天喝　2) □每周喝　3) □很少喝　4) □从不喝　5) □已戒酒

7. 您使用下列方法清洁牙齿吗？**(每小题选一个答案)**

	6<br>每天<br>≥2 次	5<br>每天<br>1 次	4<br>每周<br>2-6 次	3<br>每周<br>1 次	2<br>每月<br>1-3 次	1<br>很少/<br>从不
1) 刷牙	□	□	□	□	□	□
2) 牙签	□	□	□	□	□	□
3) 牙线	□	□	□	□	□	□

8. 您使用牙膏刷牙吗？**（只选一个答案）**
1) □是 2) □否 3) □不知道**（选 2 或 3 项者不回答第 9 题）**

9. 您使用含氟牙膏刷牙吗？**（只选一个答案）**
1) □是 2) □否 3) □不知道

10. 您看过牙吗？**（只选一个答案）**
1) □看过 2) □从没看过牙**（选 2 项者不回答第 11 至 15 题）**

11. 您最近一次看牙距现在多长时间？**（只选一个答案）**
1) □ 6 个月以内 2) □ 6 个月至 12 个月**（选 1 或 2 项者不回答第 16 题）**
3) □ 12 个月以上**（选 3 项者不回答第 12 至 15 题）**

12. 您最近一次看牙的主要原因是什么？**（只选一个答案）**
1) □咨询检查 2) □预防 3) □治疗 4) □不知道

13. 在过去的一年内您看牙的总费用是 ________ 元？**（请填一个整数，不知道或拒绝回答的填写"N"）**

14. 在上述看牙费用中，您个人需要支付的比例是 ________ %。**（请填一个整数，不知道或拒绝回答的填写"N"）**

15. 您上一次看牙，费用是否可报销？**（可多选）**
1) □城镇职工基本保险 2) □城镇居民基本医疗保险
3) □新型农村合作医疗 4) □商业保险
5) □公费医疗 6) □其他途径报销
7) □全部自费（没有报销）

16. 您过去 12 个月内**没有看过牙的原因**是？**（可多选）**
1) □牙齿没有问题 2) □牙病不重
3) □没有时间 4) □经济困难，看不起牙
5) □看牙不能报销 6) □附近没有牙医
7) □害怕传染病 8) □害怕看牙疼痛
9) □很难找到信得过的牙医 10) □挂号太难
11) □其它原因

17. 您是否有以下的医疗保障？**(每小题选一个答案)**

	是 1	否 2
1) 城镇职工基本医疗保险	□	□
2) 城镇居民基本医疗保险	□	□
3) 新型农村合作医疗	□	□
4) 商业保险	□	□
5) 公费医疗	□	□

18. 在过去的 12 个月内，您洗过牙吗？

1) □是 2) □否（**选 2 项者不回答第 19 题**）

19. 您洗牙费用的报销方式是（**可多选**）

1) □城镇职工基本保险 2) □城镇居民基本医疗保险
3) □新型农村合作医疗 4) □商业保险
5) □公费医疗 6) □其他途径报销
7) □全部自费（没有报销）

20. 口腔问题对您以下方面的影响有多大？ **(55-64 岁年龄组不回答此题，每小题选一个答案)**

	1<br>很经常	2<br>经常	3<br>有时	4<br>很少	5<br>无
1) 您经常因为牙齿或假牙的原因限制所吃食物的种类和数量吗？	□	□	□	□	□
2) 您在咬或咀嚼食物时有困难吗？	□	□	□	□	□
3) 您吞咽食物时经常会感到不舒服或困难吗？	□	□	□	□	□
4) 您的牙齿或假牙妨碍您说话吗？	□	□	□	□	□
5) 您吃东西时经常感到口腔内不舒服吗？	□	□	□	□	□
6) 您经常因为牙齿或假牙的原因而限制自己与他人的交往吗？	□	□	□	□	□
7) 您经常对您牙齿、牙龈或假牙的外观感到不满意或不愉快吗？	□	□	□	□	□
8) 您经常用药物缓解口腔的疼痛或不适吗？	□	□	□	□	□
9) 您经常担心或关注您的牙齿、牙龈或假牙的问题吗？	□	□	□	□	□
10)您经常因为牙齿、牙龈或假牙的问题而在别人面前感到紧张或不自在吗？	□	□	□	□	□
11)您经常因为牙齿或假牙的问题而在别人面前吃东西时感到不舒服吗？	□	□	□	□	□
12)您的牙齿或牙龈对冷、热或甜刺激敏感吗？	□	□	□	□	□

21. 您对自己的全身健康状况评价如何？（**只选一个答案**）

1) □很好 2) □较好 3) □一般
4) □较差 5) □很差

22. 您对自己的牙齿和口腔状况评价如何？（**只选一个答案**）

1) □很好 2) □较好 3) □一般
4) □较差 5) □很差

23. 您对以下说法的看法如何？**(每小题选一个答案)**

	1<br>同意	2<br>不同意	8<br>无所谓	9<br>不知道
1)口腔健康对自己的生活很重要	☐	☐	☐	☐
2)定期口腔检查是十分必要的	☐	☐	☐	☐
3)牙齿的好坏是天生的，与自己的保护关系不大	☐	☐	☐	☐
4)预防牙病首先靠自己	☐	☐	☐	☐

24. 你认为下面的说法是否正确? **(每小题选一个答案)**

	1<br>正确	2<br>不正确	8<br>不知道
1) 刷牙时牙龈出血是正常的	☐	☐	☐
2) 细菌可以引起牙龈发炎	☐	☐	☐
3) 刷牙对预防牙龈出血**没有用**	☐	☐	☐
4) 细菌可以引起龋齿	☐	☐	☐
5) 吃糖可以导致龋齿	☐	☐	☐
6) 氟化物对保护牙齿**没有用**	☐	☐	☐
7) 窝沟封闭可保护牙齿	☐	☐	☐
8) 口腔疾病可能会影响全身健康	☐	☐	☐

25. 你是否曾经患过由医生确诊过的下列慢性病？（**可多选**）

1) ☐中风　2) ☐糖尿病　3) ☐高血压
4) ☐心脏病　5) ☐慢性阻塞性肺部疾病　6) ☐其它，请注明________
7) ☐没有　8）☐不知道

26. 您家里共同生活的有几口人？________ 人（**请填一个整数，拒绝回答的填写“N”**）；

27. 您家在过去的12个月内的总收入大约是多少？_____万元/年（**请填一个整数，拒绝回答的填写“N”**）。

十分感谢您的合作！
