# Supplementary material for: Status of dental caries and associated factors in Tibetan adults: findings from the fourth China National Oral Health Survey
Source: BMC Oral Health. 2020 Sep 7;20:248. doi: 10.1186/s12903-020-01225-0 (PMC7487806; doi:10.1186/s12903-020-01225-0)
Supplement: Supplementary file 2 — Additional file 2: Supplementary file 2. Questionnaire translated into English. [file 12903_2020_1225_MOESM2_ESM.pdf]

（English translation）

The fourth National Oral Health Survey Questionnaire in China（For Adults）

Respondents ID：□□□□□□□□□□□ Respondents Name：__________

Survey date：201□Y□□M□□D Investigator No. :□

Requirement: Please tick"√" in the "□" in front of the corresponding option.

1. What is your highest educational background?（**Choose only one answer**）

1) □ Didn't go to school 2) □ Primary school 3) □Junior high school 4)□Senior high school
5) □ Vocational school 6)□ Junior college 7) □Undergraduate 8) □Postgraduate and above

2. How often do you usually eat the following food or drink? (**Choose one answer for each item**)

	6<br>≥2<br>times/day	5<br>1<br>time/day	4<br>2-6<br>times/week	3<br>1<br>time/week	2<br>1-3<br>times/month	1<br>rarely<br>/never
1) Dessert (biscuits, cakes, bread) and confectionery (chocolate, gum with sugar)	□	□	□	□	□	□
2) Sweet drinks（carbonate beverages such as sugar water, coke, orange juice, apple juice and other fruit juice、unfreshly squeezed juice such as lemonade）	□	□	□	□	□	□
3) Sweetened milk, yogurt, milk powder, tea, soybean milk, coffee	□	□	□	□	□	□

3. Do you smoke?（**Choose only one answer**）

1) □Smoking 2) □Never smoking 3) □ Smoking cessation（**If choose item 2 or 3, don't answer questions 4 and 5**）

4. How many years do you smoke? ____________year.（**Please fill in an integer, and fill in "N" if you have no idea or refuse to answer**）

5. During the past one month, how many cigarettes do you smoke one day on average?（**Choose only one answer**）

1) □ ≤1 /day 2) □ 1-5/day 3) □ 6-10/day
4) □ 11-20/day 5) □ 21-40/day 6) □ ≥41/day

6. Do you drink white liquor?（**Choose only one answer**）

1) □Drink every day 2) □ Drink every week 3) □ Drink seldom
4) □ Never drink 5) □ Have stopped drinking

7. Do you use the following method to clean your teeth?(**Choose one answer for each item**)

	6<br>≥2<br>times/day	5<br>1<br>time/day	4<br>2-6<br>times/week	3<br>1 time/week	2<br>1-3<br>times/month	1<br>rarely /<br>never
1)Brushing teeth	□	□	□	□	□	□
2) Toothpick	□	□	□	□	□	□
3) Dental floss	□	□	□	□	□	□

8. Do you use toothpaste when brush your teeth? (**Choose only one answer**)
1) ☐ Yes 2) ☐ No 3) ☐ Have no idea
(**If choose item 2 or 3, don't answer question 9**)

9. Do you use toothpaste with fluoride when brush your teeth? (**Choose only one answer**)
1) ☐ Yes 2) ☐ No 3) ☐ Have no idea

10. Have you had your teeth checked? (**Choose only one answer**)
1) ☐ Checked 2) ☐ Never be checked
(**If choose item 2, don't answer questions 11-15**)

11. How long has your last dental appointment from now? (**Choose only one answer**)
1) ☐ Within 6 months 2) ☐ 6 months to 12 months
(**If choose item 1 or 2, don't answer question 16**)
3) ☐ More than 12 months
(**If choose item 3, don't answer questions 12-15**)

12. What is the main reason for you to have your teeth checked? (**Choose only one answer**)
1) ☐ Consultation and examination 2) ☐ Prevention
3) ☐ Therapy 4) ☐ Have no idea

13. How much did you spend on dental therapy in the past year ________ yuan? (**Please fill in an integer, and fill in "N" if you have no idea or refuse to answer**)

14. How many percents of the above dental expenses did you personally have to pay ________ %. (**Please fill in an integer, and fill in "N" if you have no idea or refuse to answer**)

15. Was the cost of your last dental visit reimbursable? (**Multiple choices**)
1) ☐ Basic insurance for urban workers 2) ☐ Basic medical insurance for non-working urban residents
3) ☐ New Rural Co-operative Medical System 4) ☐ Commercial insurance
5) ☐ Free medical service 6) ☐ Reimbursement by other means
7) ☐ Self-paying (No reimbursement)

16. What were the reasons not to have your teeth checked in the past 12 months? (**Multiple choices**)
1) ☐ Nothing wrong with teeth 2) ☐ Dental disease was not serious
3) ☐ Have no time 4) ☐ Economic hardship to pay the dental visits
5) ☐ Dental visits are not reimbursable 6) ☐ Their was no dentist nearby
7) ☐ Fear of spreading disease 8) ☐ Fear of toothache
9) ☐ Difficult to find a reliable dentist 10) ☐ Difficult to registration
11) ☐ Other reasons

17. Do you have the following medical coverage?**(Choose one answer)**

	Yes1	No2
1)Basic insurance for urban workers	☐	☐
2)Basic medical insurance for non-working urban residents	☐	☐
3)New Rural Co-operative Medical System	☐	☐
4)Commercial insurance	☐	☐
5)Free medical service	☐	☐

18. Have you had your teeth cleaned in the past 12 months?

1) ☐Yes 2) ☐No

(If choose item 2, don't answer question 19)

19. What pattern of reimburse of the cost of your teeth cleaning（**Multiple choice**）

1) ☐ Basic insurance for urban workers 2) ☐ Basic medical insurance for non-working urban residents
3) ☐ New Rural Co-operative Medical System 4) ☐ Commercial insurance
5) ☐ Free medical service 6) ☐ Reimbursement by other means
7) ☐ Self-paying (No reimbursement)

20. How much do oral problems affect you in the following ways? **(55-64 age group do not need to answer this question, choose one answer for each question)**

	1<br>Very often	2<br>Often	3<br>Sometimes	4<br>Rarely	5<br>None
1) Do you often limit the type and amount of food you eat because of your teeth or dentures?	☐	☐	☐	☐	☐
2) Do you have any difficulty biting or chewing your food?	☐	☐	☐	☐	☐
3) Do you often feel uncomfortable or difficult when you swallow food?	☐	☐	☐	☐	☐
4) Are your teeth or dentures hindering you speak?	☐	☐	☐	☐	☐
5) Do you often feel uncomfortable in your mouth when you eat?	☐	☐	☐	☐	☐
6) Do you often limit your interactions with others due to teeth or dentures?	☐	☐	☐	☐	☐
7) Do you often feel dissatisfied or unhappy with the appearance of your teeth, gums or dentures?	☐	☐	☐	☐	☐
8) Do you often take medicine to relieve pain or discomfort in your mouth?	☐	☐	☐	☐	☐
9) Do you often worry or pay attention to your teeth, gums or dentures?	☐	☐	☐	☐	☐
10) Do you often feel nervous or uncomfortable in front of others because of your teeth, gums or dentures?	☐	☐	☐	☐	☐
11) Do you often feel uncomfortable when eating in front of others because of your teeth or dentures?	☐	☐	☐	☐	☐
12) Are your teeth or gums sensitive to cold, hot or sweet stimuli?	☐	☐	☐	☐	☐

21. How do you evaluate your general health?（**Choose only one answer**）

1) ☐ Very good 2) ☐ Good 3) ☐ Generally
4) ☐ Poor 5) ☐ Very poor

22. How do you estimate your teeth and oral health?（**Choose only one answer**）

1) ☐ Very good 2) ☐ Good 3) ☐ Generally
4) ☐ Poor 5) ☐ Very poor

23. What is your opinion about the following statement?**(Choose an answer for Each item)**

	1 Agree	2 Disagree	8 Doesn't matter	9 Have no idea
1) Oral health is very important to your life	☐	☐	☐	☐
2) Regular oral check is very necessary	☐	☐	☐	☐
3) The status of teeth are innate, has little to do with their protection	☐	☐	☐	☐
4) Prevention of dental disease is mostly by themselves	☐	☐	☐	☐

24. Do you think of the following statements are correct? **(Choose an answer for each item)**

	1 Correct	2 Incorrect	8 Have no idea
1) Gingival bleeding is normal when brushing teeth	☐	☐	☐
2) Bacteria can cause inflammation of gum	☐	☐	☐
3) Brushing teeth is useless to prevent gingival bleeding	☐	☐	☐
4) Bacteria can cause dental caries	☐	☐	☐
5) Sugar can cause dental caries	☐	☐	☐
6) Fluoride is useless to protect teeth	☐	☐	☐
7) Pit and fissure sealing can protect teeth	☐	☐	☐
8) Oral disease may affect the body health	☐	☐	☐

25. Have you ever been diagnosed the following chronic diseases by the doctor?（**Multiple choice**）

1) ☐Stroke 2) ☐Diabetes 3) ☐Hypertension
4) ☐Heart disease 5) ☐Chronic obstructive pulmonary disease 6) ☐Other, please specify ____
7) ☐None 8) ☐Have no idea

26. How many people are there in your family together? ________（**Please fill in an integer, and fill in "N" if you have no idea or refuse to answer**）;

27. The total income of your family in the past 12 months? _____ Ten thousand yuan/year（**Please fill in an integer, and fill in "N" if you have no idea or refuse to answer**）**.**

Thank you very much for your cooperation!
